# Production and analysis of multiply charged negative ions by liquid atmospheric pressure matrix‐assisted laser desorption/ionization mass spectrometry

**DOI:** 10.1002/rcm.8246

**Published:** 2018-09-12

**Authors:** Oliver J. Hale, Pavel Ryumin, Jeffery M. Brown, Michael Morris, Rainer Cramer

**Affiliations:** ^1^ Department of Chemistry University of Reading Whiteknights Reading RG6 6AD UK; ^2^ Waters Corporation Stamford Avenue Wilmslow SK9 4AX UK

## Abstract

**Rationale:**

Liquid atmospheric pressure matrix‐assisted laser desorption/ionisation (AP‐MALDI) has been shown to enable the production of electrospray ionisation (ESI)‐like multiply charged analyte ions with little sample consumption and long‐lasting, robust ion yield for sensitive analysis by mass spectrometry (MS). Previous reports have focused on positive ion production. Here, we report an initial optimisation of liquid AP‐MALDI for ESI‐like negative ion production and its application to the analysis of peptides/proteins, DNA and lipids.

**Methods:**

The instrumentation employed for this study is identical to that of earlier liquid AP‐MALDI MS studies for positive analyte ion production with a simple non‐commercial AP ion source that is attached to a Waters Synapt G2‐Si mass spectrometer and incorporates a heated ion transfer tube. The preparation of liquid MALDI matrices is similar to positive ion mode analysis but has been adjusted for negative ion mode by changing the chromophore to 3‐aminoquinoline and 9‐aminoacridine for further improvements.

**Results:**

For DNA, liquid AP‐MALDI MS analysis benefited from switching to 9‐aminoacridine‐based MALDI samples and the negative ion mode, increasing the number of charges by up to a factor of 2 and the analyte ion signal intensities by more than 10‐fold compared with the positive ion mode. The limit of detection was recorded at around 10 fmol for ATGCAT. For lipids, negative ion mode analysis provided a fully orthogonal set of detected lipids.

**Conclusions:**

Negative ion mode is a sensitive alternative to positive ion mode in liquid AP‐MALDI MS analysis. In particular, the analysis of lipids and DNA benefited from the complementarity of the detected lipid species and the vastly greater DNA ion signal intensities in negative ion mode.

## 1 INTRODUCTION

Substantial advances have recently been made with respect to the production of electrospray ionisation (ESI)‐like multiply charged (bio)macromolecules such as peptides and proteins using non‐ESI techniques,^1^, ^2^ including laser‐based desorption techniques.^3^, ^4^ With regard to the latter techniques, most of these, if not all, have been achieved and reported for the analysis of positive analyte ions. In particular, liquid atmospheric pressure matrix‐assisted laser desorption/ionisation (AP‐MALDI) has been shown to produce multiply charged positive ion species while also providing the advantage of low sample consumption and high analytical sensitivity.^3^, ^5^ Examples of potential application areas include bottom‐up proteomics^6^, ^7^ with the advantages of off‐line sample preparation, high‐speed mass spectrometry (MS) analysis, long‐term sample storage and higher tolerance to trifluoroacetic acid (TFA) when compared with ESI, and the analysis of lipids,^8^ enabling new fragment ion measurements, and, thus, the detection of diagnostic ions for elucidating fatty acid double‐bond locations even from crude biofluids like milk. The latter was achieved by employing an extremely simple sample preparation method and conventional collision‐induced dissociation (CID) and in‐source fragmentation as implemented on standard commercial instrumentation. Many of the advantages described above have not been demonstrated with any other laser‐based ionisation technique while producing multiply charged analyte ions at the same time. In addition, one of the main and unique advantages of liquid MALDI is its extremely stable ion production and sample longevity, enabling analyte ion production for hours or tens of thousands of laser shots from as little as 1 μL of sample with very little analyte ion signal variability and sample consumption.^9^, ^10^, ^11^ Using an AP heated ion source, this extremely high ion signal stability and low sample consumption can also be achieved for the production of predominantly multiply charged ESI‐like analyte ions.^3^, ^7^

The mechanisms behind the production of multiply charged ions using laser‐based desorption techniques and the link to ESI have been discussed in previous publications.^5^, ^12^, ^13^ Although many future application areas have been explored for liquid AP‐MALDI MS in the positive ion mode, no studies have explored its potential by switching to the negative ion mode. Here, it should be emphasised that liquid AP‐MALDI MS should not be confused with MALDI MS employing ionic liquid matrices in conventional vacuum ion sources as the latter still employs solid MALDI samples in many cases and does not lead to ESI‐like charge states.

This contribution presents an exploratory study of the differences and potential advantages of employing the negative ion mode in liquid AP‐MALDI MS as used for the production of ESI‐like negatively charged biomolecules. The focus will be on three classes of molecules. Using peptides and proteins, overall liquid matrix optimisation was undertaken as well as a comparison of the detection response between a basic and acidic protein in both the positive and negative ion mode. Second, DNA was studied as an obvious analyte class with respect to the potential gain by switching to the negative ion mode. The benefits of negative DNA ion analysis have been recorded in many articles, including some of the first articles published on DNA analysis using conventional solid MALDI MS.^14^, ^15^, ^16^ Third, lipids from simple milk extracts were investigated with respect to the complementary information gained by switching to the negative ion mode. Previous studies on MALDI MS analyses of lipids in the negative ion mode have pointed out the opportunities of detecting different types of lipids compared with the positive ion mode.^17^, ^18^ Although liquid AP‐MALDI does not increase the charge state of lipids as it does for larger biomolecules, its simple sample preparation is highly convenient for the MS analysis of crude liquids and their constituents, providing an extremely stable ion beam.

## 2 EXPERIMENTAL

Water, methanol, acetonitrile, isopropanol and trifluoroacetic acid (TFA) (all HPLC‐grade) were bought from Fisher Scientific (Loughborough, UK). HPLC‐grade hexane and all other matrix components were purchased from Sigma‐Aldrich (Gillingham, UK). The single‐stranded DNA/oligodeoxyribonucleotides (aqueous 200‐pmol/μL solutions) and the lyophilised protein standards bovine ubiquitin (≥98%) and trypsin inhibitor from soybean were also purchased from Sigma‐Aldrich while the peptide standard mixture (Peptide Calibration Standard II) was bought from Bruker UK (Coventry, UK), containing the peptides bradykinin 1–7, angiotensin II, angiotensin I, substance P, bombesin, ACTH clip 1–17, ACTH clip 18–39, and somatostatin 28. Analyte solutions were made up by dissolving these standards in water at 10 pmol/μL unless otherwise stated. The peptide standard mixture was prepared as per the manufacturer's instructions. The lipids extract from milk was obtained by a one‐pot two‐phase extraction method by adding 450 μL of a hexane/isopropanol mixture (3:2; v/v) to 50 μL of bovine whole milk from a local supermarket. The mixture was vortexed and centrifuged for 2 min at 13,000 rpm at room temperature. The supernatant was then used as analyte solution without further purification or concentration.

The 2,5‐dihydroxybenzoic acid (DHB)‐based matrix solution was obtained by preparing 100 μL of a 25‐mg/mL DHB solution using 10mM ammonium phosphate and acetonitrile in a ratio of 3:7 (v/v) followed by the addition of 60 μL of ethylene glycol and thorough mixing. The α‐cyano‐4‐hydroxycinnamic acid (CHCA)‐, 3‐aminoquinoline (3‐AQ)‐ and 9‐aminoacridine (9‐AA)‐based matrix solutions were similarly prepared but at a lower chromophore concentration of 5 mg/mL (CHCA) and 10 mg/mL (3‐AQ and 9‐AA) in the initial 100‐μL solution. The CHCA/3‐AQ‐based matrix solution was obtained by mixing CHCA, 3‐AQ, 10mM ammonium phosphate/methanol (1:1; v/v), and ethylene glycol in the ratio of 1:3:5:5 (w [mg]/w [mg]/v [μL]/v [μL]), applying vigorous mixing and temperatures up to 40°C.

Liquid MALDI samples were prepared in triplicates by spotting 500 nL of matrix solution on a commercial stainless steel MALDI target and adding another 500 nL of analyte solution at three different positions on the MALDI target plate.

MS data acquisition was performed on a Synapt G2‐Si HDMS (Waters, Wilmslow, UK) instrument in positive and negative ion mobility time‐of‐flight (TOF) MS modes. ‘Sensitivity' ion optics were selected. The instrument was modified using a custom‐built AP‐MALDI ion source the details of which have been described elsewhere.^5^ The ion source was operated under standard settings with the potential between the MALDI target and the ion transfer tube at 3–3.5 kV and the cone voltage at 30–40 V. The NiCr resistance wire (~6 Ω) around the ion transfer tube was heated up by approx. 29 W delivered by a low‐voltage DC power supply. Counter‐flow N_2_ gas was provided through the heated ion transfer tube up to a rate of 200 L/h. Without counter‐flow gas the temperature in the ion transfer tube was around 100° C while it was around 200° C at 180–200 L/h counter flow.^5^ The laser was an MNL100 nitrogen laser (LTB Lasertechnik Berlin, Berlin, Germany) and operated at a repetition rate of 30 Hz with approximately 22 μJ/pulse. The drift gas in the travelling wave ion mobility spectrometry (TWIMS) cell was N_2_. Experiments in the *m/z* range of 100–5000 had a pusher interval of 110 μs. Lipid spectra were acquired in the *m/z* range of 100–2000 and a pusher interval of 69 μs.

In both ion modes TOF calibration was performed by AP‐LDI with a droplet containing 500 nL of a sodium iodide solution (2 μg/μL in H_2_O/IPA 1:1; v/v) and 500 nL of a solution of acetonitrile/water/ethylene glycol at a ratio of 7:3:6 (v/v/v) spotted and mixed on the MALDI target. Calibration data was acquired by focusing the laser beam on the droplet's edge and acquiring for ≥3 min in the *m/z* range of 100–5000. Automatic peak detection and mass spectral calibration were performed in MassLynx 4.1/Intellistart (Waters). The TWIMS cell of the instrument was calibrated with polyalanine to allow determination of collision cross sections (CCSs) as required.

Mass spectra were processed with MassLynx 4.1. Where ion signal intensity is reported, the spectra were first subjected to MassLynx' automatic peak detection and analysis with automatic smoothing and centroiding. Protein spectra were processed with UniDec (University of Oxford, Oxford, UK), which was primarily used for binning and background subtraction.^19^ DriftScope version 2.8 (Waters Corp.) was used to view and extract ion mobility MS data. Progenesis QI (version 2.3; Waters Corp.) was used to search the LIPID MAPS structural database^20^ and Waters Metabolic Profiling CCS library (Waters Corporation) with tolerances of ±10 ppm for mass accuracy and ± 2% for CCS (applicable to the latter database only). Additional CCS values following the same criteria are cited from the literature.^21^

## 3 RESULTS AND DISCUSSION

In the first set of experiments, two established liquid MALDI sample preparations from earlier positive ion mode studies were used as well as two preparations that were slightly different by swapping the matrix chromophores with compounds which promised to be more suitable for negative ion mode analysis. The former two preparations employed DHB and CHCA/3‐AQ, respectively, while the latter two employed 3‐AQ (without CHCA) and 9‐AA as the chromophores; otherwise the MALDI sample preparations were identical.

In positive ion mode it was evident that out of the four different liquid MALDI sample preparations the one with DHB outperformed all of the other three with respect to the highest mean signal intensity for virtually all peptides and charge states (see Table 1). In fact, many charge states, particularly 3+, 4+ and 5+, were only detected with the DHB‐based liquid matrix. Interestingly, in negative ion mode multiply charged peptide ions were far less observed (mainly for the ACTH clips) and no ion signal at all was obtained for somatostatin (see Table 2). This poor performance was somewhat surprising since instrument parameters were tuned for negative ion mode prior to data acquisition. However, the two peptides substance P and bombesin were detected, although they have no acidic sites (not even at the C‐terminus) that could readily deprotonate. More importantly, substantially lower negative ion signal intensities have previously been reported for basic peptides like the ones used here.^22^ Contrary to the positive ion mode, the liquid MALDI matrices containing 3‐AQ (without CHCA) and 9‐AA performed best in producing singly charged negative peptide ions. The 9‐AA‐based MALDI sample droplet produced the greatest ion signal for the doubly and triply charged ACTH clip 18–39. The latter was also the only triply charged negative peptide ion signal amongst all matrices. Both 3‐AQ and 9‐AA are well‐known matrices for the negative ion mode in solid MALDI MS,^23^, ^24^ whose beneficial MALDI properties have been suggested to be due to their high gas‐phase basicities relative to the analyte.^25^

**TABLE 1:**
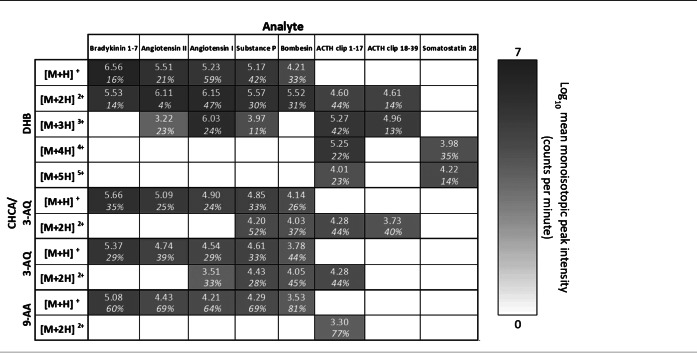
Mean MS signal intensities of centroided monoisotopic peaks of positive peptide ions for liquid MALDI samples (*n* = 3) with various matrix chromophores

The relative standard deviation (RSD%) is reported in italics.

**TABLE 2:**
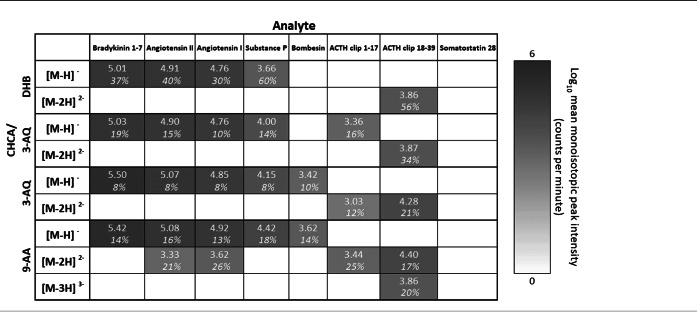
Mean MS signal intensities of centroided monoisotopic peaks of negative peptide ions for liquid MALDI samples (n = 3) with various matrix chromophores

The relative standard deviation (RSD%) is reported in italics.

**FIGURE 1:**
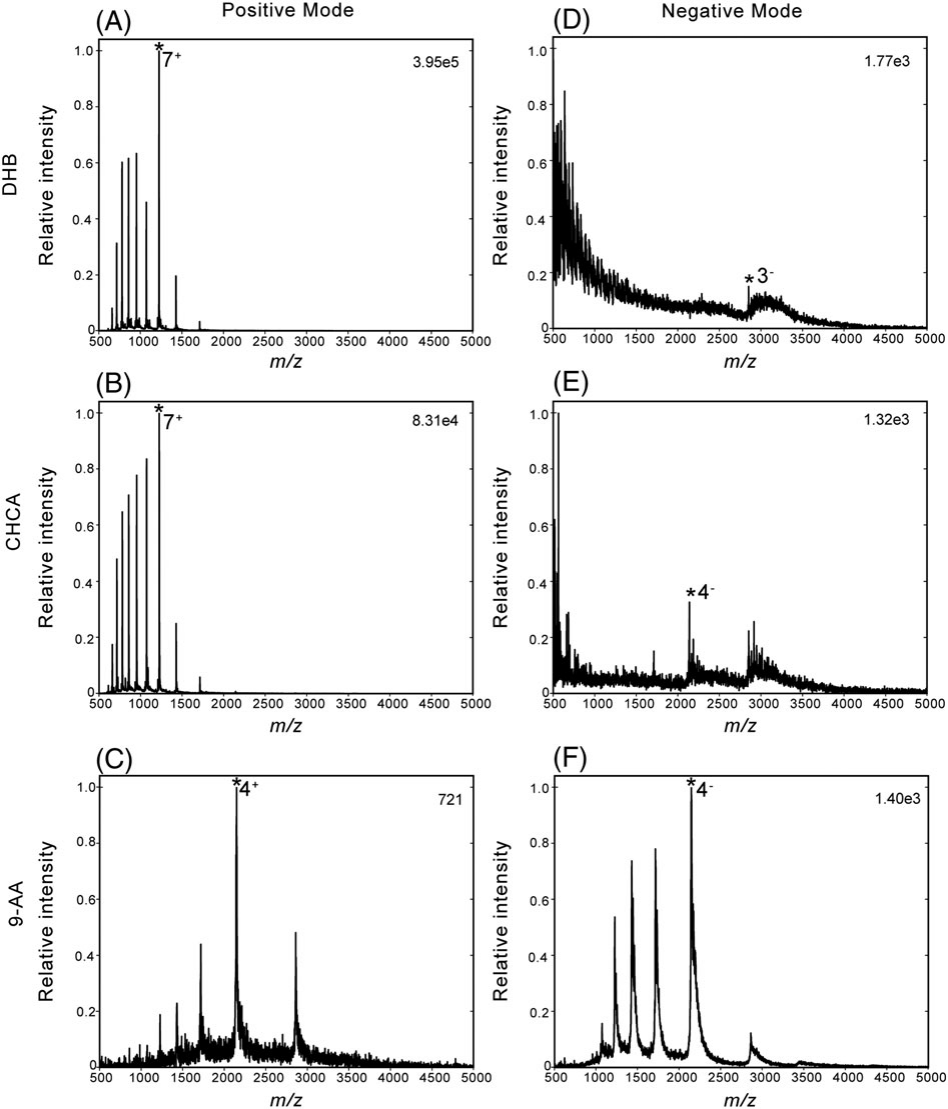
Liquid AP‐MALDI‐Q‐TOF MS spectra of ubiquitin analysed using three different matrix chromophores in positive A‐C, and negative D‐F, ion modes: DHB A‐D, CHCA B‐E, and 9‐AA C‐F. The data was acquired over 1 min at a laser pulse repetition rate of 10 Hz. The bin size was set to *m/z* 1 with minimum background subtraction. The most abundant charge state is marked by an asterisk

The same four liquid matrices used for peptide analysis were also investigated for protein analysis. In addition, a fifth liquid matrix that was prepared with CHCA as the only chromophore was also tested, since this was previously found to be superior to the DHB‐based liquid matrix for the detection of larger proteins.^12^ For ubiquitin, both the CHCA‐ and DHB‐based liquid MALDI samples performed best with respect to positive ion signal intensities. In negative ion mode, the best performing liquid MALDI samples were clearly the ones containing 9‐AA while the DHB‐, CHCA‐ and CHCA/3‐AQ‐based MALDI samples hardly produced any negative protein ions (see Figure 1). Interestingly, for ubiquitin in the positive ion mode, the recorded protein ion charge states for the DHB‐ and CHCA‐based samples covered a range of 5–14, displaying a bimodal distribution in the case of DHB. In all other cases the charge state distribution covered a smaller and lower range of 3–7, which was similar to the range when negative protein ion signals were obtained.

As ubiquitin is a basic protein with an isoelectric point of around 8.5, another protein, trypsin inhibitor, with an isoelectric point of ≤5 was also analysed. Commercial trypsin inhibitor is typically provided as a mixture of three different proteoforms at low purity. In addition, its molecular weight is almost three times as much as that of ubiquitin. Therefore, compared with ubiquitin the overall quality of the mass spectra was lower, i.e. lower protein ion signal intensities amongst higher background ion signals. As anticipated the CHCA‐based liquid MALDI samples now outperformed the DHB‐based liquid MALDI samples with respect to ion signal intensity in positive ion mode. In negative ion mode, neither of these two liquid matrices facilitated the detection of trypsin inhibitor while the 9‐AA‐based liquid MALDI samples allowed its detection in both ion modes albeit at a much‐reduced signal intensity (see Figure S1, supporting information).

For both proteins 9‐AA provided substantially higher ion signal intensities in the negative ion mode. However, in all cases the negative ion mode resulted in much higher adduct ion formation, which was identified as the addition of sodium in the cases where the resolution and signal intensity were sufficiently high.

The analyses of these two proteins using matrix chromophores with substantially different gas‐phase basicities (proton affinities) indicate stronger negative ion signals for liquid MALDI samples employing chromophores with greater gas‐phase basicity. On the other hand, matrix chromophores with lower gas‐phase basicity (lower proton affinity) result in virtually no negative protein ion signals but substantial positive protein ion signals, with the lowest proton affinity (CHCA) leading to the greatest positive ion signal intensities. A similar correlation has been exploited in previous research, designing new solid MALDI matrices such as 4‐chloro‐α‐cyanocinnamic acid,^26^ outperforming the traditional matrices DHB and CHCA, which was also observed by their counterparts in earlier work of liquid MALDI MS analysis of peptides.^11^ Interestingly, DHB has always been preferred to CHCA for the analysis of proteins in solid MALDI, arguably due to its greater ‘softness'. In liquid MALDI MS, other aspects such as proton affinity might have greater influence as this and previously published data suggest.^12^

**FIGURE 2:**
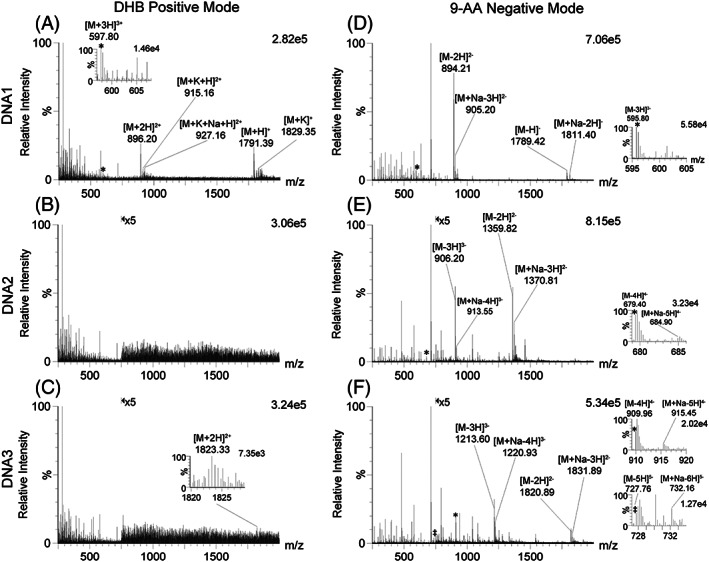
Centroided liquid AP‐MALDI‐QTOF MS spectra using DHB‐based liquid MALDI samples in positive ion mode A‐C, and 9‐AA‐based liquid MALDI samples in negative ion mode D‐F: DNA1 A‐D, DNA2 B‐E, and DNA3 C‐F. The data was acquired over 1 min at a laser pulse repetition rate of 10 Hz. For b, c, e and f, the ion signal intensity is magnified by a factor of 5 for the ions above *m/z* 750 as indicated in the top of the spectra. Insets show zoom‐ins for some of the multiply charged analyte ions

Another group of analytes that was investigated consisted of three single‐stranded DNA/oligodeoxyribonucleotide molecules of various length. Their sequences were ATGCAT (DNA1), ATGCATGCA (DNA2), and ATGCATGCATGC (DNA3). As can be seen in Figure S2 (see supporting information) positive ion signals were generally weak and the DHB‐ and CHCA/3‐AQ‐based matrices clearly outperformed the basic chromophores 9‐AA and 3‐AQ. In negative ion mode, once more the latter two resulted in far greater ion signal intensities compared with the former, with a clear tendency to produce higher levels of multiply charged ions. For ESI MS analysis of oligonucleotides, negative ion mode generally outperforms positive ion mode.^27^ Thus, given the apparent commonalities with ESI processes, it is not surprising that this was also observed for liquid AP‐MALDI MS using basic chromophores. In particular, the MALDI samples using 9‐AA showed extremely strong negative ion signals, which were superior to the positive ion signals of all tested MALDI samples with respect to the number of charges, the individual DNA ion signal intensities (apart from the singly charged ion signal of the short DNA1 analyte) and the overall DNA ion signal intensity summed up over all charged states. Comparing the spectra with the greatest DNA ion signal intensities for the positive and negative ion mode, i.e. DHB‐based samples in positive ion mode *vs* 9‐AA‐based samples in negative ion mode, respectively, the difference in spectral quality and signal‐to‐noise becomes evident (see Figure 2). It appears that in the positive ion mode cation adducts are produced with a preference for potassium adduct ion formation (Figure 2A) while sodium adduct ions were observed in the negative ion mode (Figures 2D–2F). In general, cation adduct formation was lower the higher the charge state was, again favouring the negative ion mode.

**FIGURE 3:**
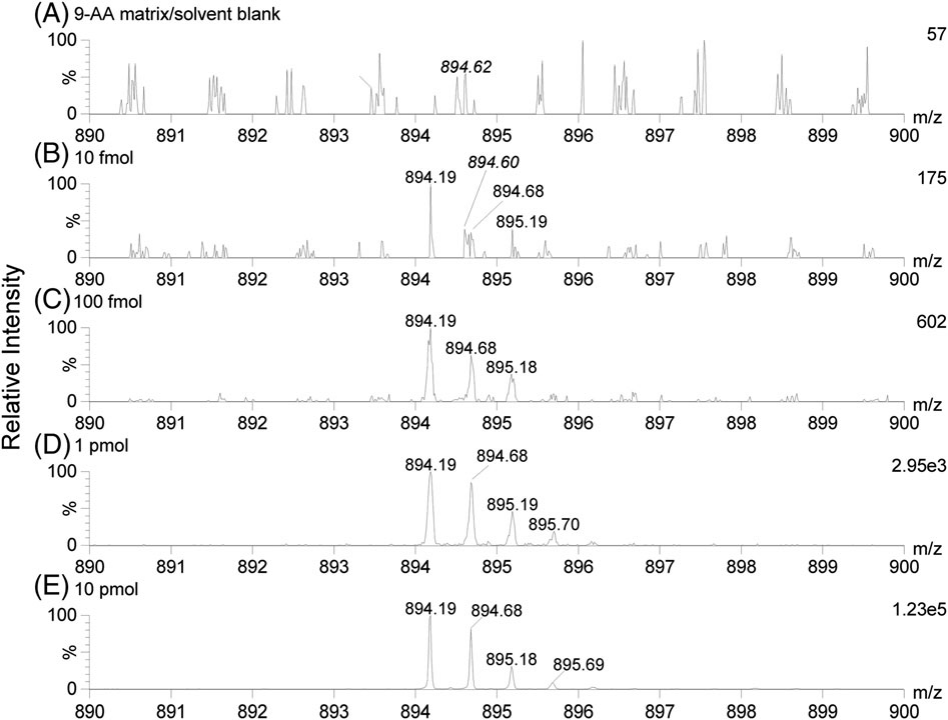
Unprocessed liquid AP‐MALDI‐Q‐TOF MS spectra indicating the limit of detection for DNA1 [M − 2H]^2−^ (*m/z* 894.19). The absolute amount per sample droplet can be found in the top left corner of the spectra. The data was acquired over 1 min at a laser pulse repetition rate of 10 Hz. In all cases only a fraction of the sample was consumed during the 1‐min acquisition. Italic *m/z* values mark ion signals not related to the analyte

For DNA analysis in the negative ion mode the limit of detection (LOD) was around 10 fmol for the doubly charged DNA1 analyte using 9‐AA as the chromophore (see Figure 3).

**FIGURE 4:**
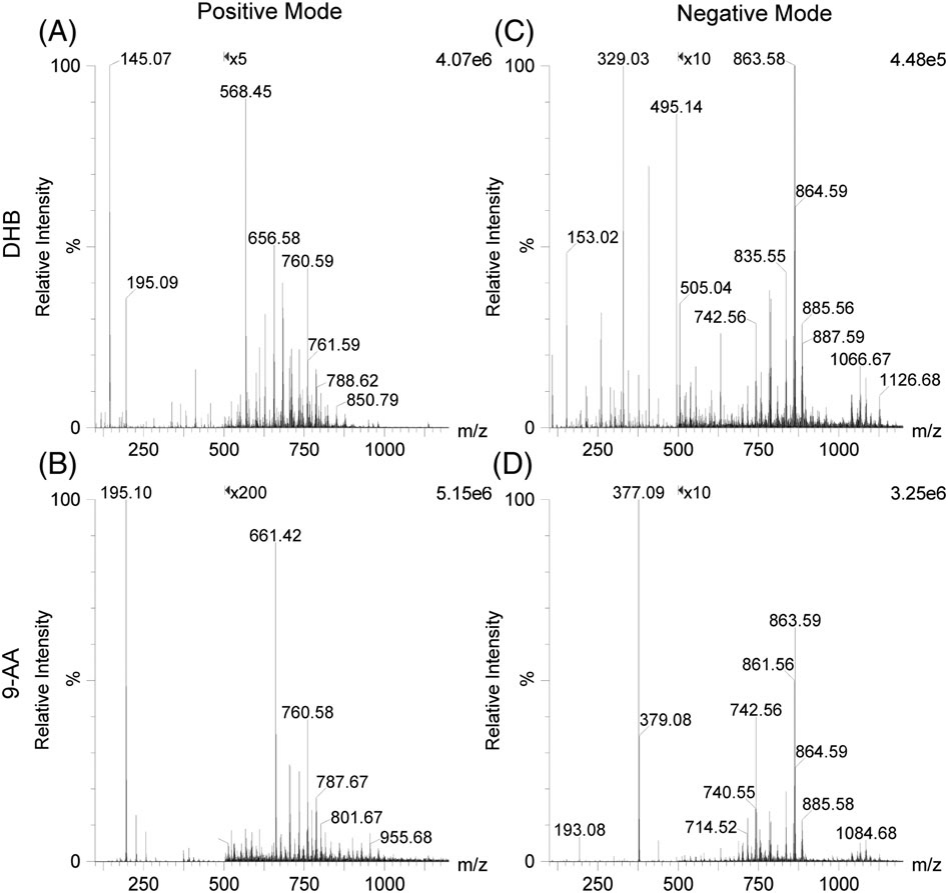
Liquid AP‐MALDI‐Q‐TOF MS spectra of lipid extracts from bovine whole milk analysed in positive A, B, and negative C, D, ion mode using DHB A, C, and 9‐AA B, D, as MALDI matrix chromophore. The ion signal intensity is magnified for the ions above *m/z* 500 as indicated in the top of the spectra. Phospholipid [M + H]^+^ and [M − H]^−^ ions putatively identified from these spectra can be found in Table S1 (supporting information)

The final analyte class tested were lipids, which were extracted from milk. The extract of milk lipids was first analysed in positive ion mode, using the above liquid MALDI sample preparation, and thus similar to the methods of previously published work,^8^ though without the addition of metal salts. As before, the DHB‐based MALDI samples provided the highest analyte ion signal intensities in positive ion mode. In negative ion mode, the 9‐AA‐based MALDI samples proved again to be the superior choice for obtaining the strongest analyte ion signal. These samples resulted in spectra with the lowest chemical background noise due to MALDI matrix ion signals (Figure 4). Advantageously, the spectra from the positive and negative ion mode are highly complementary, and thus the good analytical performance of liquid MALDI in negative ion mode is a great benefit, able to add an additional dimension of diagnostic information. Table S1 (supporting information) details the putatively identified lipids in positive and negative ion mode for the DHB‐ and 9‐AA‐based liquid MALDI samples, demonstrating the complementarity of the data without any overlap of the assigned lipid ions between the two ion modes. It is evident that phosphatidylcholines (PC) and/or isobaric phosphatidylethanolamines (PE) were mostly detected in positive ion mode while phosphatidylserines (PS) were mostly detected in negative ion mode. However, ceramides (Cer) and sphingomyelins (SM) were only detected in positive ion mode while phosphatidylinositols (PI) were only detected in negative ion mode. Phosphatidylglycerols (PG), phosphatidic acids (PA) and other lipids with variable substituents (e.g. plasmalogen) were recorded in both ion modes.

Overall, negative ion mode liquid AP‐MALDI MS shows a very similar ionisation behaviour compared with positive ion mode liquid AP‐MALDI MS. This is not too surprising if a charge‐providing MALDI matrix chromophore and an ESI‐like process for the final ionisation step are considered. Thus, similar ESI processes and differences between negative and positive ion mode analysis can be expected. Aspects of these have been discussed previously for liquid AP‐MALDI and other laser‐based ionisation techniques. For liquid AP‐MALDI, factors such as the sample surface tension and the type of matrix seem to have an important influence on the ionisation.^12^ However, in many cases, the use of small concentrations of additives, e.g. other matrix compounds or acids such as TFA, do not seem to influence the overall analytical performance, allowing greater flexibility in sample composition than what is possible with ESI.^7^, ^28^

The data of the work presented here suggest that the type of matrix and its basicity, and thus the overall basicity of the liquid MALDI sample, is arguably another factor that needs further consideration and future investigations for establishing optimal liquid MALDI sample preparations, in particular for analytes that might benefit from negative ion mode analysis.

Data supporting the results reported in this paper are openly available from the University of Reading Research Data Archive at https://doi.org/10.17864/1947.144.

## 4 CONCLUSIONS

Mass spectrometry analysis in negative ion mode is frequently inferior to positive ion mode analysis, which can be partly attributed to the mass and mobility of the electrons (compared with protons and other cations) and the technical challenges arising from this in the negative ion mode. However, for many analytes and MS sample environments/conditions, negative ion mode analysis has its benefits. In this study, data has been collected for liquid MALDI MS, producing multiply charged analyte ions in both ion modes, that is in general agreement with this difference between negative and positive ion mode analysis. While no real benefit was found for the analysis of peptides and proteins by switching to negative ion mode analysis, the analysis of other analytes was significantly enhanced by the negative ion mode, in particular in combination with a switch to a different MALDI matrix chromophore such as 9‐aminoacridine with a higher gas‐phase basicity. The liquid MALDI MS analysis of DNA in negative ion mode showed significantly better performance with regard to the individual DNA ion signal intensities and the overall DNA ion signal intensity summed up over all charged states, enabling the detection of higher charge states that were not detectable in the positive ion mode. This improvement was particularly evident for larger DNA. So far the recorded limit of detection (LOD) is around 10 fmol, using low nanomolar concentrations for MALDI sample preparation. Arguably, the greatest benefit of negative ion mode analysis was found in the analysis of lipid extracts from milk, where the two ion modes provided highly complementary datasets of lipids without any overlap.

## Supporting information

Figure S1: Liquid AP‐MALDI‐Q‐TOF MS spectra of trypsin inhibitor (Mw ~21,000 Da) analysed using three different matrix chromophores in positive (a‐c) and negative (d‐f) ion modes: DHB (a, d), CHCA (b, e) and 9‐AA (c, f). The data was acquired over 1 minute at a laser pulse repetition rate of 10 Hz. The bin size was set to *m/z* 1 with minimum background subtraction. The most abundant charge state is marked by an asterisk
Figure S2: Centroided liquid AP‐MALDI‐Q‐TOF MS spectra (positive ion mode) of DNA1 using four different matrix chromophores: (a) DHB, (b) CHCA/3‐AQ, (c) 3‐AQ and (d) 9‐AA. The data was acquired over 1 minute at a laser pulse repetition rate of 10 Hz. The ion signal intensity is magnified for the ions above *m/z* 500 as indicated in the top of the spectra.
Table S1: Identified milk lipids from liquid AP‐MALDI MS and ion mobility data
