# Supplementary material for: Production and analysis of multiply charged negative ions by liquid atmospheric pressure matrix‐assisted laser desorption/ionization mass spectrometry
Source: Rapid Commun Mass Spectrom. 2018 Sep 12;35(Suppl 1):e8246. doi: 10.1002/rcm.8246 (PMC7757204; doi:10.1002/rcm.8246)
Supplement: Supplementary file 1 — Figure S1: Liquid AP‐MALDI‐Q‐TOF MS spectra of trypsin inhibitor (Mw ~21,000 Da) analysed using three different matrix chromophores in positive (a‐c) and negative (d‐f) ion modes: DHB (a, d), CHCA (b, e) and 9‐AA (c, f). The data was acquired over 1 minute at a laser pulse repetition rate of 10 Hz. The bin size was set to m/z 1 with minimum background subtraction. The most abundant charge state is marked by an asterisk Figure S2: Centroided liquid AP‐MALDI‐Q‐TOF MS spectra (positive ion mode) of DNA1 using four different matrix chromophores: (a) DHB, (b) CHCA/3‐AQ, (c) 3‐AQ and (d) 9‐AA. The data was acquired over 1 minute at a laser pulse repetition rate of 10 Hz. The ion signal intensity is magnified for the ions above m/z 500 as indicated in the top of the spectra. Table S1: Identified milk lipids from liquid AP‐MALDI MS and ion mobility data [file RCM-35-e8246-s001.pdf]

Production and analysis of multiply charged negative ions by liquid AP-MALDI MS

Supporting Information

Oliver J. Hale[1], Pavel Ryumin[1], Jeff Brown[1,2], Michael Morris[2] and Rainer Cramer[1*]

[1] Department of Chemistry, University of Reading, Whiteknights, Reading, RG6 6AD, UK

[2] Waters Corporation, Stamford Avenue, Wilmslow, SK9 4AX, UK

***Corresponding author:**

Prof. Rainer Cramer

Department of Chemistry

University of Reading

Whiteknights

Reading RG6 6AD

UK

Email: r.k.cramer@reading.ac.uk

phone: +44 118 378 4550

fax: +44 118 378 6331

Figure S1

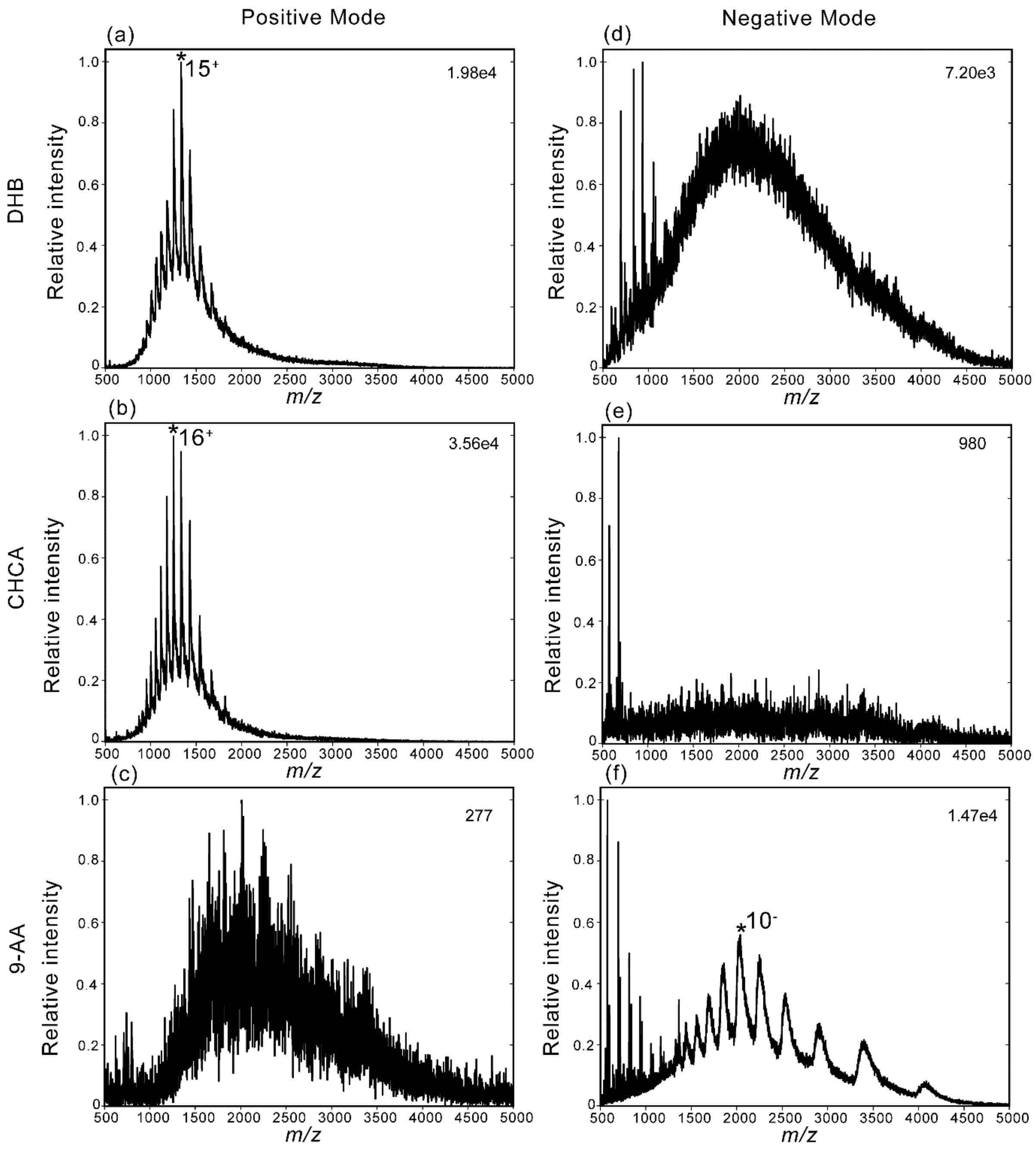

Liquid AP-MALDI-Q-TOF MS spectra of trypsin inhibitor (Mw ~21,000 Da) analysed using three different matrix chromophores in positive (a-c) and negative (d-f) ion modes: DHB (a, d), CHCA (b, e) and 9-AA (c, f). The data was acquired over 1 minute at a laser pulse repetition rate of 10 Hz. The bin size was set to m/z 1 with minimum background subtraction. The most abundant charge state is marked by an asterisk

Figure S2

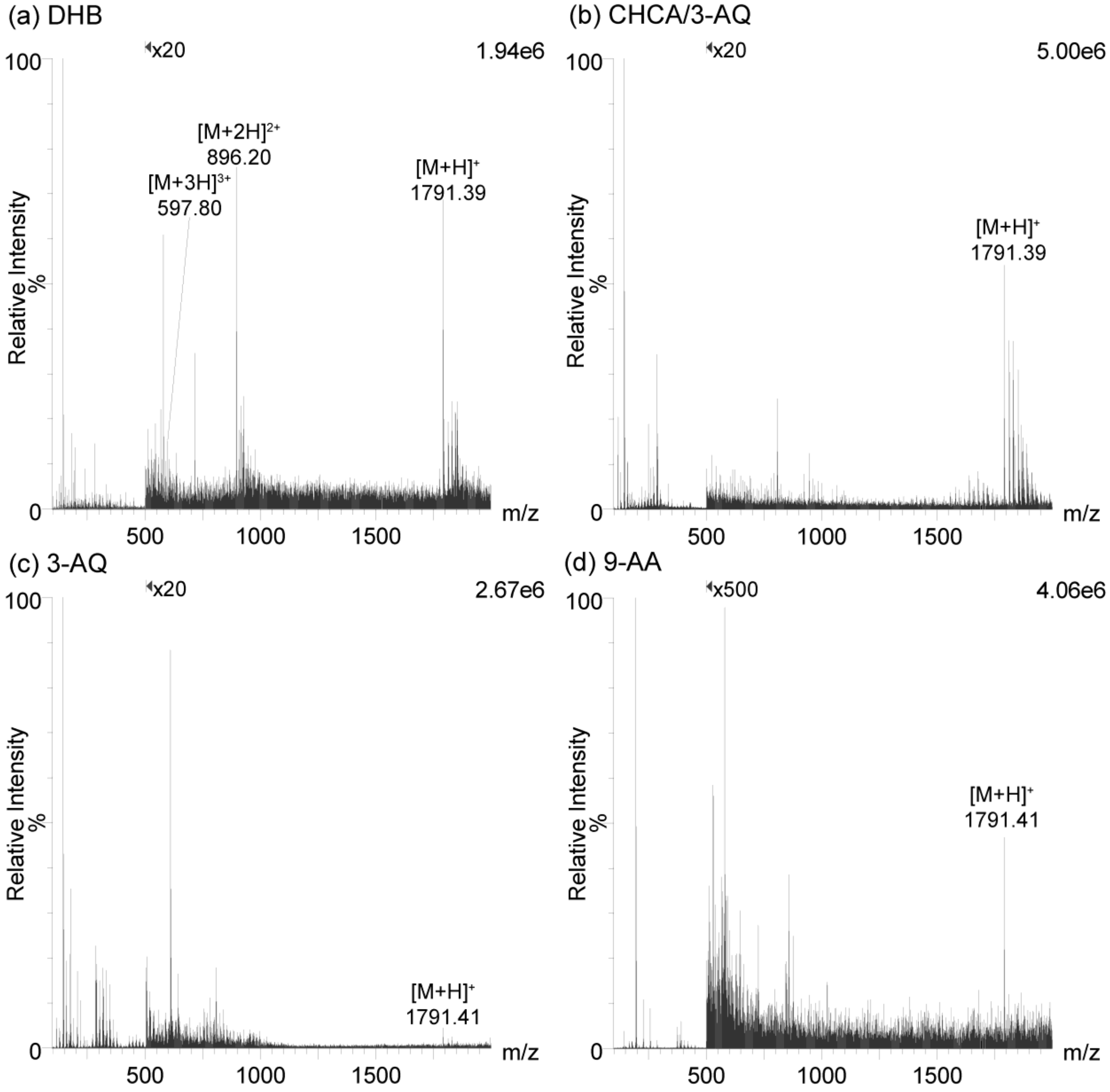

Centroided liquid AP-MALDI-Q-TOF MS spectra (positive ion mode) of DNA1 using four different matrix chromophores: (a) DHB, (b) CHCA/3-AQ, (c) 3-AQ and (d) 9-AA. The data was acquired over 1 minute at a laser pulse repetition rate of 10 Hz. The ion signal intensity is magnified for the ions above m/z 500 as indicated in the top of the spectra.

Table S1

Identified milk lipids from liquid AP-MALDI MS and ion mobility data

Description[a]	m/z	Mass Error (ppm)	Ion	CCS (Å²)[b]	∆CCS (%)[c]	Ref. CCS (Å²)[d]
**Cer(d40:1(2OH))**	638.605	-4.7	M+H			
**PC(25:0(CHO))**	650.439	0.4	M+H			
**PA(O-34:1)**	661.520	4.5	M+H			
**PA(P-34:0)**	661.520	4.5	M+H			
**PS(P-28:0)**	664.456	2.4	M+H			
**PA(34:5)**	665.425	9.3	M-H			
**SM(d32:1)**	675.539	-6.7	M+H			
**PE-Cer(d35:1)**	675.539	-6.7	M+H			
**PE-Cer(d34:1(2OH))**	677.523	0.3	M+H			
**PS(28:0)**	678.430	-7.6	M-H			
**PA(35:5)**	679.431	-5.5	M-H			
**PG(O-30:1)**	679.494	4.5	M+H			
**PG(P-30:0)**	679.494	4.5	M+H			
**SM(d16:1/17:0)**	689.555	-6.3	M+H			
**PE-Cer(d36:1)**	689.555	-6.3	M+H			
**PS(P-30:1)**	690.475	5.9	M+H			
**PI(24:0)**	697.391	-4.1	M-H			
**PA(O-37:2)**	699.533	-0.3	M-H			
**PA(P-37:1)**	699.533	-0.3	M-H			
**PA(P-37:0)**	701.546	-4.6	M-H			
**SM(d34:1)**	703.573	-2.5	M+H	293	0.8%	291
**PE-Cer(d37:1)**	703.573	-2.5	M+H			
**PG(31:1)**	705.469	-2.6	M-H			
**PA(O-37:0)**	705.581	2.2	M+H			
**PC(30:0)**	706.538	-0.4	M+H			
**PE-NMe(32:0)**	706.538	-0.4	M+H			
**PE(33:0)**	706.538	-0.4	M+H			
**PA(O-38:1)**	717.586	9.2	M+H			
**PA(P-38:0)**	717.586	9.2	M+H			
**SM(d35:1)**	717.586	-6.5	M+H			
**PE-Cer(d38:1)**	717.586	-6.5	M+H			
**PC(31:1)**	718.545	9.5	M+H			
**PE(34:1)**	718.545	9.5	M+H			
**PC(31:0)**	720.553	-1.5	M+H			
**PE-NMe2(32:0)**	720.553	-1.5	M+H			
**PE(34:0)**	720.553	-1.5	M+H			
**PI(26:0)**	725.432	9.5	M-H			
**PC(32:3)**	728.518	-6.8	M+H			
**PE(35:3)**	728.518	-6.8	M+H			
**SM(d36:1)**	731.600	-8.8	M+H	299	0.7%	297
**PA(O-39:1)**	731.600	6.6	M+H			
**PA(P-39:0)**	731.600	6.6	M+H			
**PE-Cer(d39:1)**	731.600	-8.8	M+H			
**PC(32:1)**	732.554	0.6	M+H	291	1.4%	287
**PE-NMe(34:1)**	732.554	0.6	M+H			
**PE(35:1)**	732.554	0.6	M+H			
**PC(32:0)**	734.569	-1.2	M+H	295	1.2%	291
**PE(35:0)**	734.569	-1.2	M+H			
**PI(37:0)**	739.438	-2.5	M-H			
**PA(40:10))**	739.438	5.4	M-H			
**PC(O-34:2)**	742.571	-5.7	M-H			
**PC(P-24:1)**	742.571	-5.7	M-H			
**PE(O-37:2)**	742.571	-5.7	M-H			
**PnC(34:1)**	742.571	-5.7	M-H			
**PG(34:3)**	745.506	5.8	M+H			

**PC(33:1)**	746.571	2.7	M+H			
**PE(36:1)**	746.571	2.7	M+H			
**PE-NMe2(34:1)**	746.571	2.7	M+H			
**PA(39:0)**	747.584	-7.8	M+H			
**PC(33:0)**	748.586	1.8	M+H			
**PE(36:0)**	748.586	1.8	M+H			
**PI(28:1)**	751.446	7.5	M-H			
**PI(28:0)**	753.454	-2.6	M-H			
**PPA(34:1)**	753.454	8.3	M-H			
**PC(34:3)**	756.549	-6.3	M+H	297	1.3%	293
**PE(37:3)**	756.549	-6.3	M+H			
**PC(34:2)**	758.565	-5.3	M+H	294	0.5%	293
**PE-NMe(36:2)**	758.565	-5.3	M+H			
**PE(27:2)**	758.565	-5.3	M+H			
**SM(d38:1)**	759.630	-9.4	M+H	306	0.2%	305
**PA(P-41:0)**	759.630	5.4	M+H			
**PC(34:1)**	760.585	0.1	M+H	298	0.9%	295
**PE(37:1)**	760.585	0.1	M+H			
**PG(35:1)**	761.539	6.7	M-H			
**PC(34:0)**	762.599	-2.4	M+H	300	1.1%	297
**PE(37:0)**	762.599	-2.4	M+H			
**PE-NMe(36:0)**	762.599	-2.4	M+H			
**SM(d39:1)**	773.652	-1.8	M+H			
**PA(O-42:0)**	775.663	6.6	M+H			
**PI(30:0)**	781.493	6.9	M-H			
**PC(36:4)**	782.566	-5.0	M+H	299	0.4%	298
**PE(39:4)**	782.566	-5.0	M+H			
**PC(36:3)**	784.577	-9.8	M+H	300	0.3%	299
**PS(O-37:2)**	786.565	-1.2	M-H			
**PS(P-37:1)**	786.565	-1.2	M-H			
**PC(36:2)**	786.598	-3.1	M+H	301	0.0%	301
**PE(39:2)**	786.598	-3.1	M+H			
**SM(d40:1)**	787.667	-2.3	M+H	311	0.1%	311
**PS(O-37:1)**	788.579	-3.1	M-H			
**PS(P-37:0)**	788.579	-3.1	M-H			
**PC(O-37:1)**	788.659	8.1	M+H			
**PC(P-37:0)**	788.659	8.1	M+H			
**PE(O-40:1)**	788.659	8.1	M+H			
**PE(P-40:0)**	788.659	8.1	M+H			
**PC(O-38:6)**	790.581	6.2	M-H			
**PC(P-38:5)**	790.581	6.2	M-H			
**PC(O-37:0)**	790.670	2.4	M+H			
**PE(O-40:0)**	790.670	2.4	M+H			
**MGDG(38:8)**	799.542	8.5	M+H			
**PG(38:4)**	799.542	-7.7	M+H			
**SM(d41:2)**	799.661	-9.6	M+H			
**PS(37:3)**	800.546	3.4	M+H			
**SM(d41:1(OH))**	801.683	-1.8	M+H	314	0.4%	313
**PI(O-33:1)**	807.543	4.6	M-H			
**PI(P-33:0)**	807.543	4.6	M-H			
**PI(O-33:0)**	809.553	-2.3	M-H			
**PA(44:3)**	811.614	-9.4	M+H			
**SM(d42:1)**	815.698	-2.7	M+H	317	0.4%	316
**PG(39:1)**	817.593	-4.3	M-H			
**PI(O-34:0)**	823.569	-1.4	M-H			
**PS(O-40:4)**	826.593	-3.6	M+H			
**PI(O-35:2)**	833.560	6.4	M-H			
**PI(O-16:0/19:1(9Z))**	835.570	-1.1	M-H			
**PI(P-35:0)**	835.570	-1.1	M-H			
**PI(O-35:0)**	837.584	-2.6	M-H			
**PG(41:1)**	845.626	-1.9	M-H			
**PG(42:6)**	849.573	9.1	M-H			
**PI(O-36:0)**	851.595	-8.0	M-H			

**PI(P-37:2)**	859.569	-1.4	M-H			
**PI (36:2)**	861.556	7.6	M-H	300	0.2%	300
**PI(O-37:2)**	861.587	1.3	M-H			
**PI(P-37:1)**	861.587	1.3	M-H			
**PI (36:1)**	863.570	5.5	M-H	306	0.6%	304
**PI(O-37:1)**	863.602	0.5	M-H			
**PI(P-37:0)**	863.602	0.5	M-H			
**PS (40:3)**	864.577	1.3	M-H	302	-1.1%	305
**PG(44:7)**	875.575	-7.0	M-H			
**PG(44:6)**	877.591	-6.7	M-H			
**PI(P-39:1)**	889.625	8.7	M-H			

[a]Lipid nomenclature adapted from the LIPID MAPS Classification System (E. Fahy, S. Subramaniam, R. Murphy, M. Nishijima, C. Raetz, T. Shimizu, F. Spener, G. van Meer, M. Wakelam, and E. Dennis, *Journal of Lipid Research,* **2009**, *50*, S9-S14). [b]Experimental collisional cross section (CCS) as measured by ion mobility. [c]Percentage difference of experimental CCS compared to the reference literature CCS. [d]Reference literature value of CCS obtained from Waters Metabolic Profiling CCS Library (Waters Corporation) or K. M. Hines, J. C. May, J. A. McLean, L. B. Xu, *Anal. Chem.* **2016**, *88*, 7329. Negative ions are highlighted in grey.
